# 1-[(*E*)-2-Formyl-1-(4-methyl­phen­yl)ethen­yl]-3-(4-methyl­phen­yl)pyrazole-4-carbaldehyde

**DOI:** 10.1107/S1600536808027694

**Published:** 2008-09-06

**Authors:** P. Ramesh, A. Subbiahpandi, Ramaiyan Manikannan, S. Muthusubramanian, M. N. Ponnuswamy

**Affiliations:** a Department of Physics, Presidency College (Autonomous), Chennai 600 005, India; b Department of Organic Chemistry, School of Chemistry, Madurai Kamaraj University, Madurai 625 021, India; c Centre of Advanced Study in Crystallography and Biophysics, University of Madras, Guindy Campus, Chennai 600 025, India

## Abstract

In the crystal structure of the title compound, C_21_H_18_N_2_O_2_, mol­ecules are linked through C—H⋯O inter­actions. Two symmetry-related mol­ecules form a cyclic centrosymmetric *R*_2_^2^(20) dimer. These dimers are further connected into chains running along the *b* axis.

## Related literature

For related literature, see: Baraldi *et al.* (1998 ▶); Bernstein *et al.* (1995 ▶); Bruno *et al.* (1990 ▶); Chen & Li (1998 ▶); Cottineau *et al.* (2002 ▶); Londershausen (1996 ▶); Mishra *et al.* (1998 ▶); Smith *et al.* (2001 ▶).
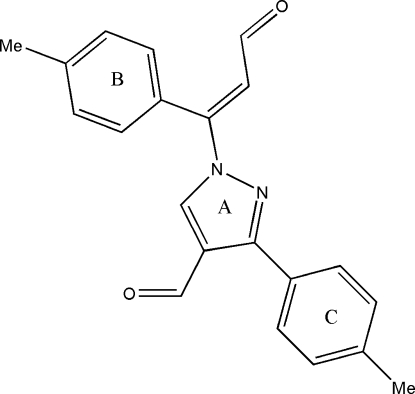


## Experimental

### 

#### Crystal data

C_21_H_18_N_2_O_2_
*M* *_r_* = 330.37
Monoclinic, 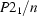

*a* = 10.2914 (4) Å
*b* = 15.3618 (5) Å
*c* = 11.0271 (4) Å
β = 98.778 (1)°
*V* = 1722.90 (11) Å^3^
*Z* = 4
Mo *K*α radiation
μ = 0.08 mm^−1^
*T* = 293 (2) K
0.30 × 0.20 × 0.16 mm

#### Data collection

Bruker APEXII CCD area-detector diffractometer
Absorption correction: multi-scan (*SADABS*; Sheldrick, 2001 ▶) *T*_min_ = 0.980, *T*_max_ = 0.987
24747 measured reflections
6019 independent reflections
3731 reflections with *I* > 2σ(*I*)
*R*_int_ = 0.025

#### Refinement

*R*[*F* ^2^ > 2σ(*F* ^2^)] = 0.055
*wR*(*F* ^2^) = 0.174
*S* = 1.04
6019 reflections
228 parameters
H-atom parameters constrained
Δρ_max_ = 0.35 e Å^−3^
Δρ_min_ = −0.30 e Å^−3^

### 

Data collection: *APEX2* (Bruker, 2004 ▶); cell refinement: *APEX2*; data reduction: *SAINT* (Bruker, 2004 ▶); program(s) used to solve structure: *SHELXS97* (Sheldrick, 2008 ▶); program(s) used to refine structure: *SHELXL97* (Sheldrick, 2008 ▶); molecular graphics: *ORTEP-3* (Farrugia, 1997 ▶); software used to prepare material for publication: *SHELXL97* and *PLATON* (Spek, 2003 ▶).

## Supplementary Material

Crystal structure: contains datablocks global, I. DOI: 10.1107/S1600536808027694/bt2774sup1.cif

Structure factors: contains datablocks I. DOI: 10.1107/S1600536808027694/bt2774Isup2.hkl

Additional supplementary materials:  crystallographic information; 3D view; checkCIF report

## Figures and Tables

**Table 1:** Hydrogen-bond geometry (Å, °)

*D*—H⋯*A*	*D*—H	H⋯*A*	*D*⋯*A*	*D*—H⋯*A*
C22—H22*A*⋯O2^i^	0.96	2.60	3.378 (2)	139
C5—H5⋯O1^ii^	0.93	2.23	3.1094 (17)	159

Symmetry codes: (i) 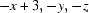; (ii) 
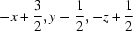
.

**Fig. 1.**
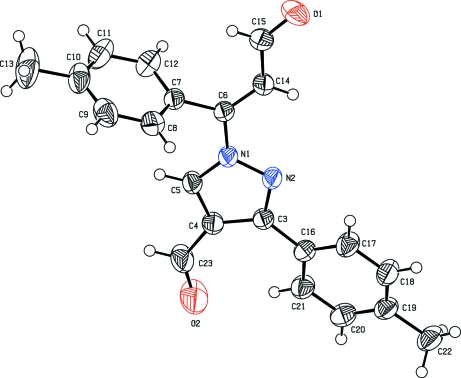
Perspective view of the molecules showing the thermal ellipsoids are drawn at 50% probability level. The H atoms are shown as small circles of arbitrary radii.

**Fig. 2.**
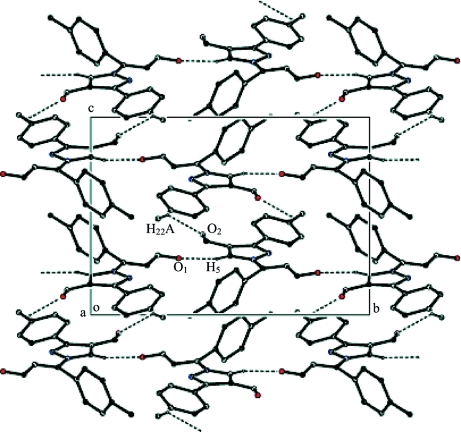
Perspective view of the crystal packing showing hydrogen-bond interactions (dashed lines). H atoms not involved in hydrogen bonding have been omitted for clarity.

## supplementary crystallographic information

### Comment

Pyrazoles and its derivatives have been reported to possess significant antiarrhythmic and sedative (Bruno *et al.*, 1990), hypoglycemic (Cottineau *et al.*, 2002), antiviral (Baraldi *et al.*, 1998), and pesticidal (Londershausen,1996) activities. Some of their derivatives have also been successfully tested for their antifungal (Chen & Li, 1998), antihistaminic (Mishra *et al.*, 1998) and anti-inflammatory (Smith *et al.*, 2001) properties.

The pyrazole ring A and methylphenyl ring C are near-coplanar with the inter-ring dihedral angle of 17.08 (7)°, whereas the pyrazole ring is twisted from the methylphenyl ring B as can be seen from the dihedral angle of 79.70 (8)°. The propenal group assumes an extended conformation which is evidenced from the torsion angle of [N1—C6—C14—C15] -175.47 (12)°.

The crystal structure is stabilized by C—H···O type of intermolecular interactions. Atom C5 at (*x*, *y*, *z*) donates a proton to atom O1 at (3/2 - *x*,-1/2 + *y*,1/2 - *z*) form a one dimensional C7 chain (Bernstein *et al.* 1995) running along *b* axis. The molecules at positions (*x*, *y*, *z*) and (3 - *x*, -*y*, -*z*) form a cyclic centrosymmetric *R*_2_^2^(20) dimer through C22—H22A···O2 hydrogen bonds.

### Experimental

A mixture of 1-(4-methylphenyl)-1-ethanone *N*-[(*E*)-1-phenylethylidene] hydrazone (0.003 mole) and 3 ml of dimethyl formamide kept in an ice bath at 0° C, phosphorus oxycholride (0.024 mole) was added dropwise for 5–10 minutes. The reaction mixture was then kept in a microwave oven at 600 W for 30–60 sec. The process of the reaction was monitored by TLC. After completion of the reaction, the reaction mixture was poured into crushed ice and extracted with dichloromethane. The organic layer was dried with anhydrous sodium sulfate. The different compounds present in the mixture were separated by column chromatography using petroleum ether and ethyl acetate mixture as eluent. This isolated compound was rectystalized in dichloromethane to obtain 3-(4-methylphenyl)-1-[(*E*)-1-(4-methylphenyl) -3-oxo-1-propenyl]-1*H*-pyrazole-4-carbaldehyde in 57% yield.

### Refinement

H atoms were positioned geometrically (C—H=0.93–0.96 Å) and allowed to ride on their parent atoms, with *U*_iso_(H) = 1.5*U*_eq_(C) for methyl H 1.2*U*_eq_(C) for other H atoms. The methyl groups were allowed to rotate but not to tip.

### Crystal data

**Table:**

C_21_H_18_N_2_O_2_	*F*(000) = 696
*M**_r_* = 330.37	*D*_x_ = 1.274 Mg m^−^^3^
Monoclinic, *P*2_1_/*n*	Mo *K*α radiation, λ = 0.71073 Å
Hall symbol: -P 2yn	Cell parameters from 4580 reflections
*a* = 10.2914 (4) Å	θ = 2.3–32.2°
*b* = 15.3618 (5) Å	µ = 0.08 mm^−^^1^
*c* = 11.0271 (4) Å	*T* = 293 K
β = 98.778 (1)°	Block, colorless
*V* = 1722.90 (11) Å^3^	0.30 × 0.20 × 0.16 mm
*Z* = 4	

### Data collection

**Table:**

Bruker APEX2 CCD area-detector diffractometer	6019 independent reflections
Radiation source: fine-focus sealed tube	3731 reflections with *I* > 2σ(*I*)
graphite	*R*_int_ = 0.025
ω and φ scans	θ_max_ = 32.3°, θ_min_ = 2.3°
Absorption correction: multi-scan (SADABS; Sheldrick, 2001)	*h* = −9→15
*T*_min_ = 0.980, *T*_max_ = 0.987	*k* = −22→19
24747 measured reflections	*l* = −16→16

### Refinement

**Table:**

Refinement on *F*^2^	Primary atom site location: structure-invariant direct methods
Least-squares matrix: full	Secondary atom site location: difference Fourier map
*R*[*F*^2^ > 2σ(*F*^2^)] = 0.055	Hydrogen site location: inferred from neighbouring sites
*wR*(*F*^2^) = 0.174	H-atom parameters constrained
*S* = 1.04	*w* = 1/[σ^2^(*F*_o_^2^) + (0.0833*P*)^2^ + 0.2417*P*] where *P* = (*F*_o_^2^ + 2*F*_c_^2^)/3
6019 reflections	(Δ/σ)_max_ = 0.037
228 parameters	Δρ_max_ = 0.35 e Å^−^^3^
0 restraints	Δρ_min_ = −0.30 e Å^−^^3^

### Special details

**Table:**

Geometry. All e.s.d.'s (except the e.s.d. in the dihedral angle between two l.s. planes) are estimated using the full covariance matrix. The cell e.s.d.'s are taken into account individually in the estimation of e.s.d.'s in distances, angles and torsion angles; correlations between e.s.d.'s in cell parameters are only used when they are defined by crystal symmetry. An approximate (isotropic) treatment of cell e.s.d.'s is used for estimating e.s.d.'s involving l.s. planes.
Refinement. Refinement of *F*^2^ against ALL reflections. The weighted *R*-factor *wR* and goodness of fit *S* are based on *F*^2^, conventional *R*-factors *R* are based on *F*, with *F* set to zero for negative *F*^2^. The threshold expression of *F*^2^ > σ(*F*^2^) is used only for calculating *R*-factors(gt) *etc*. and is not relevant to the choice of reflections for refinement. *R*-factors based on *F*^2^ are statistically about twice as large as those based on *F*, and *R*- factors based on ALL data will be even larger.

### Fractional atomic coordinates and isotropic or equivalent isotropic displacement parameters (Å^2^)

**Table:**

	*x*	*y*	*z*	*U*_iso_*/*U*_eq_	
O1	0.65358 (11)	0.31462 (7)	0.28639 (13)	0.0678 (3)	
O2	1.23915 (18)	−0.09914 (9)	0.0928 (2)	0.1340 (9)	
N1	0.93785 (9)	0.08384 (7)	0.22048 (10)	0.0378 (2)	
N2	1.03155 (10)	0.13820 (7)	0.18711 (10)	0.0395 (2)	
C3	1.12006 (11)	0.08674 (8)	0.14811 (11)	0.0367 (3)	
C4	1.08139 (12)	−0.00258 (8)	0.15437 (12)	0.0413 (3)	
C5	0.96509 (12)	−0.00004 (8)	0.20099 (12)	0.0401 (3)	
H5	0.9144	−0.0478	0.2163	0.048*	
C6	0.82656 (11)	0.11747 (8)	0.26494 (11)	0.0367 (3)	
C7	0.75369 (11)	0.05214 (8)	0.32683 (12)	0.0381 (3)	
C8	0.80708 (14)	0.01954 (10)	0.44021 (14)	0.0509 (3)	
H8	0.8884	0.0396	0.4784	0.061*	
C9	0.74082 (17)	−0.04269 (11)	0.49748 (15)	0.0593 (4)	
H9	0.7777	−0.0632	0.5744	0.071*	
C10	0.62160 (17)	−0.07477 (10)	0.44295 (16)	0.0594 (4)	
C11	0.56858 (16)	−0.04197 (12)	0.33110 (18)	0.0648 (5)	
H11	0.4876	−0.0627	0.2931	0.078*	
C12	0.63277 (14)	0.02175 (11)	0.27265 (14)	0.0527 (4)	
H12	0.5940	0.0437	0.1971	0.063*	
C13	0.5526 (2)	−0.14496 (13)	0.5044 (2)	0.0956 (8)	
H13A	0.4601	−0.1432	0.4741	0.143*	
H13B	0.5667	−0.1356	0.5915	0.143*	
H13C	0.5872	−0.2008	0.4867	0.143*	
C14	0.79599 (12)	0.20179 (9)	0.25103 (13)	0.0438 (3)	
H14	0.8454	0.2369	0.2066	0.053*	
C15	0.68876 (13)	0.24017 (10)	0.30271 (14)	0.0487 (3)	
H15	0.6441	0.2053	0.3516	0.058*	
C16	1.23610 (11)	0.12790 (9)	0.10905 (12)	0.0386 (3)	
C17	1.26700 (13)	0.21329 (10)	0.14214 (14)	0.0497 (3)	
H17	1.2159	0.2431	0.1911	0.060*	
C18	1.37242 (14)	0.25480 (10)	0.10352 (15)	0.0527 (4)	
H18	1.3910	0.3122	0.1268	0.063*	
C19	1.45103 (12)	0.21259 (10)	0.03075 (13)	0.0454 (3)	
C20	1.42132 (13)	0.12763 (10)	−0.00071 (14)	0.0503 (3)	
H20	1.4736	0.0978	−0.0486	0.060*	
C21	1.31552 (13)	0.08512 (10)	0.03692 (13)	0.0475 (3)	
H21	1.2976	0.0276	0.0137	0.057*	
C22	1.56456 (14)	0.25876 (12)	−0.01291 (16)	0.0582 (4)	
H22A	1.6425	0.2240	0.0057	0.087*	
H22B	1.5783	0.3140	0.0277	0.087*	
H22C	1.5451	0.2677	−0.1000	0.087*	
C23	1.13601 (18)	−0.08585 (10)	0.12661 (19)	0.0684 (5)	
H23	1.0849	−0.1346	0.1362	0.082*	

### Atomic displacement parameters (Å^2^)

**Table:**

	*U*^11^	*U*^22^	*U*^33^	*U*^12^	*U*^13^	*U*^23^
O1	0.0641 (7)	0.0420 (7)	0.0968 (9)	0.0162 (5)	0.0110 (6)	−0.0076 (6)
O2	0.1215 (13)	0.0506 (8)	0.263 (2)	0.0205 (8)	0.1355 (15)	0.0071 (11)
N1	0.0371 (5)	0.0308 (5)	0.0488 (6)	0.0001 (4)	0.0169 (4)	0.0010 (4)
N2	0.0387 (5)	0.0325 (6)	0.0508 (6)	−0.0020 (4)	0.0180 (4)	0.0002 (4)
C3	0.0366 (5)	0.0355 (7)	0.0399 (6)	0.0009 (4)	0.0117 (4)	0.0008 (5)
C4	0.0424 (6)	0.0353 (7)	0.0493 (7)	0.0019 (5)	0.0171 (5)	−0.0013 (5)
C5	0.0433 (6)	0.0298 (6)	0.0502 (7)	−0.0006 (5)	0.0167 (5)	−0.0007 (5)
C6	0.0337 (5)	0.0343 (6)	0.0442 (6)	0.0010 (4)	0.0129 (5)	0.0013 (5)
C7	0.0373 (5)	0.0332 (6)	0.0470 (7)	0.0008 (4)	0.0166 (5)	0.0000 (5)
C8	0.0489 (7)	0.0486 (8)	0.0561 (8)	0.0020 (6)	0.0110 (6)	0.0086 (6)
C9	0.0766 (10)	0.0492 (9)	0.0572 (9)	0.0087 (8)	0.0266 (8)	0.0136 (7)
C10	0.0757 (10)	0.0409 (8)	0.0730 (11)	−0.0059 (7)	0.0476 (8)	−0.0042 (7)
C11	0.0556 (8)	0.0655 (11)	0.0784 (12)	−0.0242 (8)	0.0266 (8)	−0.0120 (9)
C12	0.0471 (7)	0.0578 (9)	0.0545 (8)	−0.0103 (6)	0.0115 (6)	0.0000 (7)
C13	0.1297 (19)	0.0620 (12)	0.1154 (18)	−0.0239 (12)	0.0837 (15)	−0.0010 (11)
C14	0.0411 (6)	0.0357 (7)	0.0578 (8)	0.0027 (5)	0.0176 (6)	0.0040 (6)
C15	0.0424 (6)	0.0409 (8)	0.0647 (9)	0.0053 (5)	0.0145 (6)	−0.0028 (6)
C16	0.0360 (5)	0.0412 (7)	0.0404 (6)	−0.0007 (5)	0.0113 (5)	0.0016 (5)
C17	0.0474 (7)	0.0471 (8)	0.0599 (9)	−0.0060 (6)	0.0247 (6)	−0.0076 (6)
C18	0.0500 (7)	0.0461 (8)	0.0661 (9)	−0.0105 (6)	0.0221 (7)	−0.0054 (7)
C19	0.0356 (6)	0.0564 (9)	0.0455 (7)	−0.0020 (5)	0.0102 (5)	0.0086 (6)
C20	0.0445 (7)	0.0573 (9)	0.0537 (8)	0.0025 (6)	0.0216 (6)	−0.0006 (6)
C21	0.0459 (7)	0.0447 (8)	0.0556 (8)	−0.0015 (5)	0.0198 (6)	−0.0052 (6)
C22	0.0425 (7)	0.0709 (11)	0.0644 (9)	−0.0070 (7)	0.0180 (6)	0.0116 (8)
C23	0.0755 (11)	0.0359 (8)	0.1052 (14)	0.0070 (7)	0.0502 (10)	0.0005 (8)

### Geometric parameters (Å, °)

**Table:**

O1—C15	1.2048 (17)	C12—H12	0.9300
O2—C23	1.1949 (19)	C13—H13A	0.9600
N1—C5	1.3429 (16)	C13—H13B	0.9600
N1—N2	1.3679 (13)	C13—H13C	0.9600
N1—C6	1.4108 (14)	C14—C15	1.4429 (17)
N2—C3	1.3263 (15)	C14—H14	0.9300
C3—C4	1.4332 (18)	C15—H15	0.9300
C3—C16	1.4726 (16)	C16—C17	1.386 (2)
C4—C5	1.3734 (16)	C16—C21	1.3896 (17)
C4—C23	1.449 (2)	C17—C18	1.3806 (18)
C5—H5	0.9300	C17—H17	0.9300
C6—C14	1.3364 (18)	C18—C19	1.384 (2)
C6—C7	1.4804 (16)	C18—H18	0.9300
C7—C12	1.3771 (19)	C19—C20	1.373 (2)
C7—C8	1.3807 (19)	C19—C22	1.5072 (17)
C8—C9	1.382 (2)	C20—C21	1.3864 (18)
C8—H8	0.9300	C20—H20	0.9300
C9—C10	1.373 (3)	C21—H21	0.9300
C9—H9	0.9300	C22—H22A	0.9600
C10—C11	1.367 (3)	C22—H22B	0.9600
C10—C13	1.507 (2)	C22—H22C	0.9600
C11—C12	1.393 (2)	C23—H23	0.9300
C11—H11	0.9300		
			
C5—N1—N2	111.71 (9)	C10—C13—H13C	109.5
C5—N1—C6	127.40 (10)	H13A—C13—H13C	109.5
N2—N1—C6	120.86 (10)	H13B—C13—H13C	109.5
C3—N2—N1	105.69 (10)	C6—C14—C15	122.12 (12)
N2—C3—C4	110.22 (10)	C6—C14—H14	118.9
N2—C3—C16	117.81 (11)	C15—C14—H14	118.9
C4—C3—C16	131.96 (11)	O1—C15—C14	124.02 (14)
C5—C4—C3	104.78 (11)	O1—C15—H15	118.0
C5—C4—C23	119.51 (13)	C14—C15—H15	118.0
C3—C4—C23	135.70 (12)	C17—C16—C21	117.85 (11)
N1—C5—C4	107.59 (11)	C17—C16—C3	119.56 (11)
N1—C5—H5	126.2	C21—C16—C3	122.58 (12)
C4—C5—H5	126.2	C18—C17—C16	121.04 (12)
C14—C6—N1	120.30 (11)	C18—C17—H17	119.5
C14—C6—C7	125.66 (10)	C16—C17—H17	119.5
N1—C6—C7	114.03 (10)	C17—C18—C19	121.24 (14)
C12—C7—C8	118.58 (12)	C17—C18—H18	119.4
C12—C7—C6	121.17 (12)	C19—C18—H18	119.4
C8—C7—C6	120.25 (11)	C20—C19—C18	117.67 (12)
C7—C8—C9	120.59 (14)	C20—C19—C22	121.35 (13)
C7—C8—H8	119.7	C18—C19—C22	120.99 (14)
C9—C8—H8	119.7	C19—C20—C21	121.80 (12)
C10—C9—C8	121.24 (15)	C19—C20—H20	119.1
C10—C9—H9	119.4	C21—C20—H20	119.1
C8—C9—H9	119.4	C20—C21—C16	120.39 (14)
C11—C10—C9	118.04 (14)	C20—C21—H21	119.8
C11—C10—C13	121.26 (18)	C16—C21—H21	119.8
C9—C10—C13	120.70 (19)	C19—C22—H22A	109.5
C10—C11—C12	121.64 (15)	C19—C22—H22B	109.5
C10—C11—H11	119.2	H22A—C22—H22B	109.5
C12—C11—H11	119.2	C19—C22—H22C	109.5
C7—C12—C11	119.90 (15)	H22A—C22—H22C	109.5
C7—C12—H12	120.1	H22B—C22—H22C	109.5
C11—C12—H12	120.1	O2—C23—C4	127.49 (16)
C10—C13—H13A	109.5	O2—C23—H23	116.3
C10—C13—H13B	109.5	C4—C23—H23	116.3
H13A—C13—H13B	109.5		
			
C5—N1—N2—C3	−1.10 (14)	C9—C10—C11—C12	0.4 (2)
C6—N1—N2—C3	−178.96 (11)	C13—C10—C11—C12	−178.80 (16)
N1—N2—C3—C4	1.07 (14)	C8—C7—C12—C11	−1.4 (2)
N1—N2—C3—C16	−178.26 (10)	C6—C7—C12—C11	177.74 (13)
N2—C3—C4—C5	−0.68 (15)	C10—C11—C12—C7	1.0 (3)
C16—C3—C4—C5	178.52 (13)	N1—C6—C14—C15	−175.47 (12)
N2—C3—C4—C23	−179.31 (18)	C7—C6—C14—C15	3.5 (2)
C16—C3—C4—C23	−0.1 (3)	C6—C14—C15—O1	−175.65 (15)
N2—N1—C5—C4	0.69 (15)	N2—C3—C16—C17	16.27 (18)
C6—N1—C5—C4	178.37 (12)	C4—C3—C16—C17	−162.88 (14)
C3—C4—C5—N1	−0.02 (15)	N2—C3—C16—C21	−162.11 (13)
C23—C4—C5—N1	178.89 (14)	C4—C3—C16—C21	18.7 (2)
C5—N1—C6—C14	−163.17 (14)	C21—C16—C17—C18	0.7 (2)
N2—N1—C6—C14	14.31 (18)	C3—C16—C17—C18	−177.75 (13)
C5—N1—C6—C7	17.72 (18)	C16—C17—C18—C19	−0.2 (2)
N2—N1—C6—C7	−164.79 (11)	C17—C18—C19—C20	−0.7 (2)
C14—C6—C7—C12	72.91 (19)	C17—C18—C19—C22	178.93 (14)
N1—C6—C7—C12	−108.04 (14)	C18—C19—C20—C21	0.9 (2)
C14—C6—C7—C8	−107.93 (16)	C22—C19—C20—C21	−178.68 (13)
N1—C6—C7—C8	71.12 (15)	C19—C20—C21—C16	−0.4 (2)
C12—C7—C8—C9	0.5 (2)	C17—C16—C21—C20	−0.5 (2)
C6—C7—C8—C9	−178.73 (13)	C3—C16—C21—C20	177.95 (12)
C7—C8—C9—C10	1.0 (2)	C5—C4—C23—O2	−175.2 (2)
C8—C9—C10—C11	−1.4 (2)	C3—C4—C23—O2	3.3 (4)
C8—C9—C10—C13	177.79 (15)		

### Hydrogen-bond geometry (Å, °)

**Table:**

*D*—H···*A*	*D*—H	H···*A*	*D*···*A*	*D*—H···*A*
C22—H22A···O2^i^	0.96	2.60	3.378 (2)	139.
C5—H5···O1^ii^	0.93	2.23	3.1094 (17)	159.

Symmetry codes: (i) −*x*+3, −*y*, −*z*; (ii) −*x*+3/2, *y*−1/2, −*z*+1/2.
